# Exploring Physicians’ Dual Perspectives on the Transition From Free Text to Structured and Standardized Documentation Practices: Interview and Participant Observational Study

**DOI:** 10.2196/63902

**Published:** 2025-03-21

**Authors:** Olga Golburean, Rune Pedersen, Line Melby, Arild Faxvaag

**Affiliations:** 1 Department of Neuromedicine and Movement Science Faculty of Medicine and Health Sciences NTNU-Norwegian University of Science and Technology Trondheim Norway; 2 Norwegian Centre for E-Health Research University Hospital of North Norway Tromsø Norway; 3 Department of Health Research SINTEF Digital Trondheim Norway

**Keywords:** documentation, documentation practice, standardized documentation, structured documentation, secondary use of data, interoperability, electronic health record, EHR

## Abstract

**Background:**

Clinical documentation plays a crucial role in providing and coordinating care. Despite the widespread adoption of electronic health record (EHR) systems, many end users still document clinical data in a manner similar to traditional paper-based records. To fully leverage the benefits of EHR systems, it is necessary to adopt new documentation approaches that facilitate easy access to information at the point of care and seamless exchange of information across health care facilities.

**Objective:**

We aimed to evaluate how the transition from an older EHR system to a cross-institutional EHR system impacts physicians’ documentation practices and gain a deeper understanding of the factors influencing their choice between free text and structured and standardized documentation methods.

**Methods:**

A qualitative study was conducted between September 2023 and January 2024. It involved participant observations and individual semistructured interviews with physicians at a university hospital in Norway. Data were analyzed using reflexive thematic analysis.

**Results:**

The analysis revealed 3 main themes. First, physicians encountered challenges during the implementation phase of the new EHR system due to its complexity and their unfamiliarity with its use. However, with time, physicians gradually adopted new documentation processes. This integration or adoption primarily occurred by learning through practical experience and collaborative knowledge exchange with their peers. Second, although the implementation of the new EHR system had increased structured and standardized clinical documentation, free text remained the preferred method, with some exceptions. In addition, the fact that many physicians still relied on free-text documentation created a sense of distrust among them toward some of the standardized clinical data. Finally, the informants had mixed perceptions of Systematized Nomenclature of Medicine–Clinical Terms. Some viewed it as a more nuanced terminology system, while others found it more complex. Most informants found using templates for routine procedures beneficial as it saved time in the documentation process and ensured that all necessary parameters and documentation requirements were met.

**Conclusions:**

The study findings revealed that physicians’ acceptance of new documentation processes is influenced by various social and technological factors. These factors include previous documentation experiences, perceived benefits, familiarity with the EHR system, time constraints, and user-friendliness of the system. While physicians generally have a positive attitude toward using templates for routine procedures, they often create their own templates, and data within these templates are documented in a free-text format. To address this, health care organizations should consider implementing common standardized or semistandardized templates to reduce disparities in documentation, enhance data recording, and ensure adherence to guidelines. Furthermore, to facilitate the transition to the new documentation processes, we recommend providing physicians with customized training programs and platforms for tacit knowledge exchange.

## Introduction

### Background

Electronic health record (EHR) systems have been advocated as means to enhance the efficiency of health care services, facilitating improved coordination and delivery of safe and evidence-based care [1,2]. Data from an EHR system can also provide health professionals with the information they need to make informed decisions at the point of care and the opportunity to reflect on their clinical work and learn from past experiences [3]. In addition, an EHR system facilitates access to data collected during clinical care, which may be used for secondary purposes (eg, research and augmenting the capabilities of clinical decision support systems) [4-6]. Nevertheless, clinical data need to be captured in a fixed structure in order to facilitate their use [4]. Health professionals play a crucial role in the documentation process, facilitating the accumulation of data in the EHR system. However, the implementation of the EHR system often results in end users still recording clinical data in a manner reminiscent of traditional paper-based records, although in a digital format, using computers and keyboards [7]. Entering structured and standardized data into an EHR system can be time-consuming, and the level of acceptance among different end users can vary greatly. Health professionals, frequently constrained by time limitations, might be reluctant to assume the data entry task unless they see significant benefits [8,9].

### Clinical Documentation in EHR Systems

Health care systems are bound by stringent laws and regulations. In Norway, the use of electronic health data for the provision of health care services (ie, the primary use of health data) is regulated by the Patient Record Act [10]. This act aims to ensure that patients receive high-quality health care by making health data available for health professionals in a quick, effective, and secure manner. Health professionals, on their side, have a duty to meet documentation requirements as outlined in the Health Personnel Act [11]. This act specifically mandates that patient journals must be maintained in accordance with high professional standards, ensuring that they include pertinent and essential information about the patient and the health care services rendered. Furthermore, the journals must be easily comprehensible for other qualified health care personnel. The use of electronic health data for purposes such as statistics, public health, health surveillance, research and product development, education, or teaching (ie, the secondary use of health data) is also strictly regulated [10,12].

The adoption of the EHR system has significantly expanded the volume of clinical data available. However, a considerable portion of these data remains underused, largely due to the dominance of unstructured documentation, such as free-text notes and narrative reports [7]. It is estimated that 80% of the EHR data exist in unstructured documents [13,14]. Consequently, there is increasing pressure on health professionals to adopt structured and standardized documentation methods [6]. This involves the use of standardized coding systems for data entry, the incorporation of models and terminologies such as Systematized Nomenclature of Medicine–Clinical Terms (SNOMED CT), and the use of structured forms [6,15]. Structured and standardized data encompass various patient details, including demographics, laboratory tests, height, weight, blood pressure, medications, allergies, and more [16,17]. Since structured and standardized data types are organized within a fixed structure, they can be easily analyzed using statistical or machine learning methods [16]. Furthermore, data from EHR systems also play a crucial role in the realization of learning health systems, where data are captured as a by-product of care. These data are subsequently analyzed to generate new knowledge, which, in turn, informs continuous improvement and innovation in health care service delivery [18,19]. While on the one hand, structured and standardized documentation practices are associated with enhanced quality of clinical notes [20,21], on the other hand, research highlights that the adoption of the EHR system has led to increased documentation time and reduced attention to patient care [6]. Moreover, structured and standardized documentation can impede expressivity in notes. Free-text documentation is crucial in health care delivery as it allows health professionals to record detailed and nuanced patient narratives. Thus, Rosenbloom et al [22] advocated that health professionals should have the flexibility to decide their documentation style according to workflow needs and content specifics. Their study highlighted that structured documentation is ideal for scenarios requiring data reuse, whereas narrative documentation is suitable when data reuse is not a priority. Furthermore, Levy et al [23] emphasized that health professionals encounter an excessive documentation burden when the usability of the documentation systems fails to adequately support patient care delivery. This highlights the crucial role of systems’ usability and the need to assess their impact on documentation practices.

The aim of this study is to evaluate how the transition from an older EHR system to a newer cross-institutional EHR system, which enables structured and standardized documentation, impacts physicians’ documentation practices. It seeks to gain insights into the factors that impact physicians’ choice of documentation methods, such as free text or structured and standardized documentation.

### Theory

Standardizing work processes in health care, such as implementing standardized guidelines for procedures and treatments, is regarded as a useful approach to improving the quality of health care due to its ability to improve outcomes, reduce the likelihood of errors, and eliminate unnecessary variations [24-26]. The increased adoption of EHR systems has led to increased interest in integrating standardized clinical tools within these systems. This includes, among others, standards pertaining to clinical documentation, encompassing what, where, and how information should be documented (eg, structured text, use of templates, and narrative text) [27-30]. Standardizing the use of the EHR system contributes to achieving interoperable EHR system, enabling the seamless exchange of clinical data among various health care organizations [31-33]. Nevertheless, the pursuit of standardization in health care, particularly in the realm of EHR systems, has proven to be challenging [31,34]. This is due to the highly heterogeneous nature of health care, marked by the constant occurrence of unforeseen circumstances. A significant portion of health care professionals’ responsibilities cannot be strictly defined by standardized procedures alone, as a substantial amount of their work is tailored to address the unique requirements of individual patients [35,36]. Thus, it has been suggested that providing flexibility is essential in health care work, as it enables health care professionals to customize their work to factors such as patient needs, good judgment, workflow, and prevailing circumstances [24]. Cherns [37] further emphasized the importance of incorporating sufficient flexibility within technical systems. This entails the ability to tailor the system according to specific local needs and adapt to evolving requirements [33,37]. This is justifiable, as Orlikowski [38] highlighted the dynamic nature of technologies, which are continually shaped by human responses to a multitude of influences, such as environmental, political, and cultural factors. Nevertheless, it is important to note that people have their own distinct viewpoints, “practice lenses” that impact how they use the systems [33,38], and may also rely on their own meanings and interpretations of the technology’s functions, known as “interpretative flexibility,” which may deviate from the designer’s original intentions [39,40]. Considering the aforementioned information, it is notable that the implementation of EHR systems and the standardized processes they encompass (eg, clinical documentation) extend beyond being mere technological tools. Instead, EHR systems are integral components of a sociotechnical system, which encompasses a dynamic interaction between technology, health professionals, organizational structures, and the social environment in which they are embedded [41-43]. Thus, literature has previously emphasized the significance of using the sociotechnical system theory [44] in the design, development, implementation, use, and evaluation of health information technology [45] and applied it to examine its impact on documentation practices [46].

### The Context

Although all health care organizations in Norway use an EHR system, many of these systems are not adequately designed for structured and standardized documentation [47]. The daily documentation primarily comprises unstructured notes written in free text, which makes it difficult to use for secondary purposes, such as quality improvements. Moreover, in many cases, medications are manually recorded and signed on paper-based charts before being scanned as images into the EHR system [47]. According to Norwegian health authorities, health professionals lack proper access to patient health information when needed at the point of care [48]. This challenge is primarily attributed to the fragmented nature of the health care systems, coupled with the insufficient integration of various information and communication technology systems [48]. Thus, in 2012, the Norwegian government launched the “One citizen—one health record” initiative. Its goal is to facilitate the seamless integration and accessibility of essential health information at every stage of patient care, regardless of when or where it is required [48]. As part of this initiative, the Health Platform (Helseplattformen) project was established [49], with the aim to replace the existing fragmented EHR systems with an integrated solution for both primary and specialist health professionals in the entire central Norway region [50,51]. The project selected Epic Systems Corporation as the vendor for the EHR system [49].

Health Platform aimed to shift toward more structured and standardized documentation of clinical data in order to achieve optimal system functionality and make data machine readable. A pivotal change within the Health Platform project is the transition from the *International Classification of Diseases, 10th Revision* (*ICD-10*) to the more comprehensive and nuanced terminology system, SNOMED CT [52]. The complexity of SNOMED CT arises from a dense hierarchical structure and “is-a” relationships that link broader and more specific concepts, as explored by Abeysinghe et al [53]. This allows SNOMED CT to capture nuances that provide better clinical coverage, making it more suitable for the EHR system and supporting patient care, compared to *ICD*, which is mainly used for diagnoses and billing [54]. The lack of detail in *ICD* codes can lead to a loss of specificity in a clinical context, which means that rare conditions can be grouped under a single code, thus impacting the EHR system’s ability to provide accurate clinical decision support [54].

## Methods

### Study Design and Settings

We used a descriptive qualitative research method to conduct this case study based on participant observations and individual semistructured interviews with physicians. Qualitative research methods enable researchers to delve deeply into the subject matter, providing comprehensive insights and detailed descriptions from the participants’ perspectives. Unlike quantitative methods, qualitative approaches, such as interviews and observations, excel at offering detailed explanations, particularly when investigating questions pertaining to *why* and *how* [35,55].

We conducted this study in 1 surgical department at a large university hospital in the central Norway region, which transitioned from a facility-centered EHR system to Health Platform, the new cross-institutional EHR system (Epic Systems Corporation), in November 2022. The university hospital houses approximately 1000 beds and employs around 11,000 staff members. The surgical department includes an outpatient clinic, inpatient ward, surgical unit, and emergency room, complemented by a specialized division for pediatrics.

### Recruitment of Participants

Participants for this study were recruited through verbal and written approaches. First, an email was sent to all physicians working within the surgical department, requesting their participation in an observational study. Second, recognizing the challenges of recruiting busy physicians, particularly during the transition to the new EHR system, the first author (OG) attended the physicians’ morning meeting to provide additional information about the project and give them the opportunity to ask direct questions about the study. The study aimed to recruit participants with varying levels of professional experience, including experienced physicians and interns, to ensure a diverse range of perspectives. After completing the observational study phase, a subsequent email invitation was sent to the same group of individuals (ie, physicians in the surgical department), inviting them to participate in individual interviews. The first author (OG) reached out to physicians interested in participating in the study to schedule a convenient time and location for their meeting. In total, 14 physicians participated in the study, and the characteristics of the participants are summarized in Table 1.

**Table 1:** Demographic characteristics of the study participants (N=14).

Characteristics			Participants, n (%)
**Age group (y)**			
	25-39	9 (64)	
	≥40	5 (36)	
**Sex**			
	Female	6 (43)	
	Male	8 (57)	
**Practice (y)**			
	<5	3 (21)	
	5-15	7 (50)	
	>15	4 (29)	

### Data Collection

#### Observations

**Figure 1:**
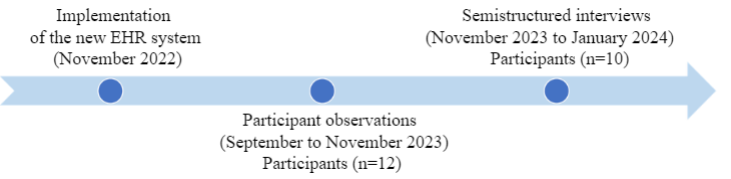
Data collection activities. EHR: electronic health record.

Participant observations occurred from September 2023 to November 2023. In total, 12 physicians were observed for a combined duration of approximately 44 hours. Figure 1 depicts the information regarding data collection activities. The observation sessions ranged from 1 to 3 times, with each session varying in duration from 1 hour to 3 hours. The observation was conducted at the surgical department at several health care facilities where the physicians were working, including an outpatient clinic, an inpatient ward, and an emergency room. Before these observations, a taxonomy was developed to guide the observational process. It was developed based on the research question, a review of existing literature [56-58], and collaborative inputs from field experts (AF, LM, and RP). The primary focus during observations was on physicians’ interactions with the new EHR system, with particular attention given to their documentation practices. Throughout each observational session, the observer (OG) recorded notes, capturing both the physicians’ activities and, when feasible, engaging them in dialogue about their EHR use. Following observations, the observer supplemented these notes with additional remarks and personal reflections.

#### Interviews

After the completion of the observations, individual, semistructured interviews were conducted face-to-face with the participants by the first author (OG). In total, 10 physicians were interviewed, 8 (80%) of whom had also been part of previous observations. The interviews were conducted between November 2023 and January 2024 (Figure 1). The interviews lasted between 34 and 60 minutes, with an average duration of 44 (SD 9.6) minutes. The interview guide, developed based on the existing literature [58-60] and expert collaboration in the field, was initially outlined before the observational phase. However, following the observations, which provided in-depth insights into the physicians’ work practices with the new EHR system, the interview guide was revised to incorporate emerging, relevant questions. Multimedia Appendix 1 provides the interview guide. Each interview was audio recorded and transcribed verbatim by the first author (OG). The process of transcription began immediately after the first interview and was integral to the preliminary data analysis phase, which involved a thorough familiarization with the collected data. The interview phase concluded once data saturation was achieved. This happened when further interviews no longer provided any new insights or themes.

### Data Analysis

**Figure 2:**
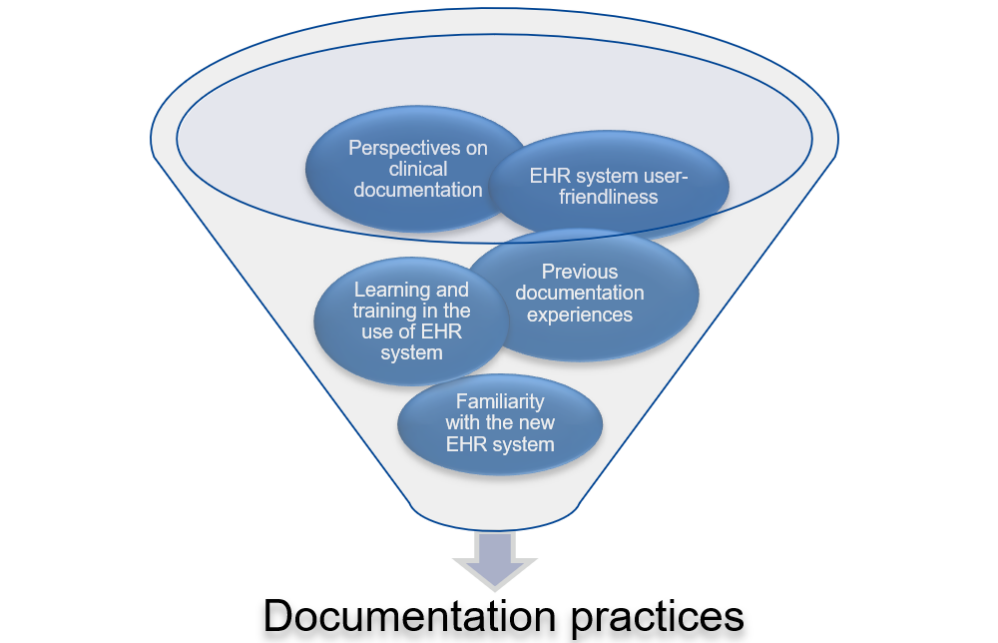
Sociotechnical factors impacting physicians’ documentation practices. EHR: electronic health record.

Data were organized using NVivo (Lumivero) software [61] and analyzed through a reflexive thematic analysis approach [62]*.* The transcripts of the interviews and field notes from the observation sessions were thoroughly examined to familiarize them with the data. Then, specific codes were developed using an inductive approach, meaning that the codes were derived from the data. The initial codes were created using a semantic coding approach. These initial codes were reviewed, leading to the formation of initial themes. Throughout this process, we continuously reviewed and revised the raw data, codes, and initial themes to ensure consistency and coherence. Moreover, we conducted a 2-day workshop where authors collaboratively reviewed the preliminary themes and their associated codes. During this workshop, we used latent analyses to gain a deeper understanding of the data within the themes. Specifically, we delved into the factors that influenced physicians’ documentation practices in the new EHR system using the sociotechnical system theory [44]. These factors encompassed various aspects, such as implications of learning and training in the use of EHR system, physicians’ perspectives and previous experiences with clinical documentation, familiarity with the new EHR system, and the influence of technology (new EHR system) and its user-friendliness on physicians’ documentation practices (Figure 2). The research team involved in the data analysis possessed diverse backgrounds that enriched the interpretation of the data. The researchers included experts in health informatics, medicine, qualitative research, and implementation science. This multidisciplinary perspective facilitated a comprehensive understanding of the complexities inherent in the data collected. Using the workshop notes, the first author (OG) further refined the themes. This phase served as the basis for drafting the initial report. The authors adhered to the SRQR (Standards for Reporting Qualitative Research) checklist [63]. Multimedia Appendix 2 provides the complete SRQR checklist.

### Ethical Considerations

This study was registered and approved by Sikt, Norwegian Agency for Shared Services in Education and Research (project number 540255). Before conducting observations and interviews, all participants were provided with written and verbal information about the study. Written consent was individually obtained from each participant. No personally identifiable data were collected, and in cases where indirectly identifiable information was mentioned during interviews, such details were omitted from all reports, papers, and presentations. Participants in this study did not receive any form of compensation.

## Results

### Overview

**Figure 3:**
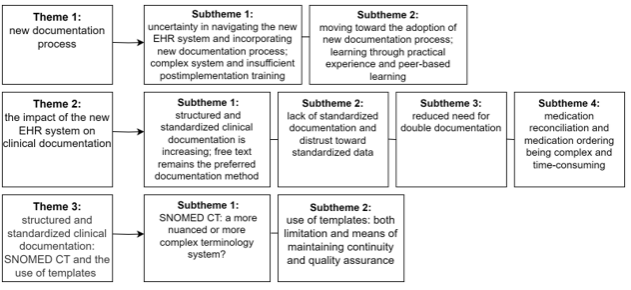
Themes and subthemes regarding clinical documentation practices emerged from the interviews and observations. EHR: electronic health record; SNOMED CT: Systematized Nomenclature of Medicine–Clinical Terms.

Through observations and interviews, 3 main themes, with corresponding subthemes related to documentation practices, were identified (Figure 3). The first theme shed light on the initial challenges faced by physicians during the implementation phase, specifically their uncertainty in navigating the new EHR system and its complexity. It also showed the gradual integration of physicians into the new processes of the EHR system, primarily by learning through practical experience and collaborative knowledge exchange with their peers (peer-based learning). The second theme contemplated the influence of the new EHR system on physicians’ documentation practices. It delved into the preferred method of documentation and provided insights into the challenges and benefits associated with the implementation of the new documentation processes. In addition, it highlighted the issue of nonstandardized documentation, which in some cases leads to a sense of distrust among physicians toward standardized clinical data. The third theme delved into the perspectives of physicians regarding the adoption of the new terminology system, SNOMED CT, and their opinions on using templates for clinical data documentation.

### New Documentation Processes

#### Uncertainty in Navigating the New EHR System and Incorporating New Documentation Processe; Complexity of the System and Insufficient Postimplementation Training

Physicians encountered a learning curve while transitioning to the new EHR system, necessitating adjustments to specific documentation practices. The complexity of certain processes and the additional administrative responsibilities linked to the new system left physicians feeling unsure about how to efficiently navigate the system and, in certain cases, how to accurately document clinical data:

... you spend more time searching for the right function that you need to document correctly, and to search for information, and then maybe you are a little bit unsure about some things, you wonder if you have included everything...there is a lot of clicking to move forward; it wasn’t like that before; it used to be more like create note, write note, done, so there are quite a lot of steps to move forward [in the new EHR]; the system controls you a bit, sort of.

Informant 10

Initially, many physicians were unfamiliar with the specific functionalities of the new EHR system and attempted to use and document clinical data in a similar manner as they did in the previous system. Consequently, certain informants encountered difficulties when using the new system due to its complexity and lack of familiarity with effective navigation:

I think that what we need now is to go through each workflow and make sure that everyone does it in the same way so that we can coordinate more, also that you can improve the shortcuts that exist and the ways of working efficiently so that everyone learns it because there are some who use the system very cumbersomely.

Informant 9

Furthermore, informants highlighted that the training they received before the implementation of the new EHR system was not sufficient. This was mainly due to substantial modifications made to the EHR system since the training and because the training did not address their specific role. In addition, some informants mentioned that differentiating between essential and less significant aspects addressed in the training became difficult if they had not yet used the system. Most informants also believed that there was insufficient personalized training provided after the implementation, and they felt that more specific and customized training was necessary to effectively use the system’s advanced features, particularly once physicians had acquired a basic understanding of the system:

We received a lot of tips and tricks in the beginning, but then it was more about doing, surviving, staying afloat, and stabilizing ourselves a bit...having new training now would have been a much bigger help at this time than it was before...

Informant 1

#### Moving Toward the Adoption of New Documentation Processes and Learning Through Practical Experience and Peer-Based Learning

On the basis of the observation sessions and interview data, it was evident that physicians were gradually adapting to new processes in the new EHR system and making changes to specific documentation practices:

When I used to admit patients, I used to write a continuous text with previous illnesses, family, and social, such as whether they lived at home or in a nursing home. I used to just list all previous illnesses, which were often taken from a previous admission journal. I have partly done it that way now as well, but there is more and more emphasis on not doing it and instead entering the information in problem lists of medical and surgical history. So, it’s a transition for me...

Informant 10

This adaptation and learning occurred both through practice (actual use of the system) and knowledge sharing among colleagues (peer-based learning). The informants found it valuable to learn from experienced colleagues. Occasionally, they also organized small group meetings where these colleagues provided guidance on navigating the system effectively. However, most physicians expressed concerns about the complexity of the newly implemented EHR system, which hindered their learning process and effective use. It was emphasized that simplifying the system and eliminating unnecessary features would greatly enhance its usability.

### The Impact of the New EHR System on Clinical Documentation

#### Structured and Standardized Clinical Documentation Is Increasing, and Free Text Remains the Preferred Documentation Method

Compared to the previous EHR system, a greater proportion of patient information was being entered in a structured and standardized format. In certain cases, the system enforced a hard stop, making it mandatory for users to input the required standardized information. For instance, in the old EHR system, the findings and diagnosis code were described in a free-text format along with codes such as *ICD-10*, whereas in the new EHR system, these aspects were documented in a standardized format using SNOMED CT. Another example was the transition from paper-based to digital documentation for ordering surgical procedures. As a result, numerous tasks were performed electronically, eliminating the reliance on paper, and more patient health information was documented in a structured and standardized format:

Well, then [previous EHR] there was nothing standardized, so...no, no, then I manually entered the procedure code and diagnosis code, and the medications were mostly just copied from one note to the next, and then you hoped that what you had copied over was correct.

Informant 6

Although structured and standardized documentation has become more prevalent, many physicians still preferred using free text whenever possible. This preference was primarily due to the flexibility and time-saving nature of free-text documentation. It was also easier for physicians to provide a comprehensive overview of a patient’s medical condition through free-text notes. Furthermore, some informants highlighted their preference for reading high-quality free-text notes as they provided a better understanding of the patient’s condition compared to information presented in templates or diagnosis codes:

The only way I manage to get an overview of the medical history of a patient is basically if someone has written something reasonable in a journal note.

Informant 4

Moreover, physicians were accustomed to free-text documentation, and transitioning to structured and standardized formats required additional time and training to ensure accuracy.

Structured and standardized documentation, on the other hand, was often perceived as time-consuming, more challenging, and sometimes misaligned with physicians’ workflows. In addition, one physician added that he did not see the benefits of adopting a more intricate approach, such as standardized documentation:

Yes, no, I need to see the benefit of doing it in a more complicated way, then I might have chosen a different way of documentation than free text. So far, I haven’t been able to see the major benefit, so that’s why I document in free text...

Informant 2

Nonetheless, some informants acknowledged that reading and finding free-text documentation could be challenging in certain situations, particularly when there were numerous notes present. In such cases, important information might become obscured or go unnoticed, whereas standardized information automatically appeared when reviewing the patient journal.

#### Lack of Standardized Documentation and Distrust Toward Standardized Data

On the basis of our findings, the preference for free-text documentation method was often accompanied by a culture of distrust among physicians toward standardized data available in the EHR system. For instance, the new EHR system integrated several processes related to clinical documentation that were not present in the previous EHR system. These included processes such as the documentation of the “problem list,” which is an overview of medical conditions that might impact the health care given to the patient, and “medical history,” which lists the conditions that have been resolved and no longer impact patients’ health or treatment. These conditions, if documented, were automatically displayed when reviewing patient journals without the need to access journal notes. However, physicians were uncertain about the accuracy of these standardized data and sometimes could not rely on it, as they were aware that many of their colleagues did not adhere to standardized documentation:

It depends on how many people enter standardized data, so I feel that I can’t rely on what is stated there. One can enter previous illnesses and medical history, but it is rarely done. So, then, the patient looks healthy on paper, but there are many illnesses, as you can find in the old journal system.

Informant 4

#### Reduced Need for Double Documentation

In certain situations, physicians favored structured and standardized documentation; for instance, in the new EHR system, they transitioned from paper-based to digital documentation when ordering surgical procedures. By adhering to structured and standardized documentation during the preoperative phase, they could save time in the postoperative phase, as some of the information was automatically filled in the postoperative template, eliminating redundant documentation. As a result, physicians recognized the benefits of using structured and standardized documentation for frequently performed routine procedures:

It is to some extent a bit easier [postoperative documentation] because all the information I enter beforehand also appears in the postoperative description afterwards, so before, I used to manually write in codes and bleeding amount, for example, but all of this is now automatically included in the postoperative description, so actually, writing the postoperative description afterwards is easier.

Informant 8

#### Medication Reconciliation and Medication Ordering Being Complex and Time-Consuming

With the implementation of the new EHR system, physicians transitioned from manually recording medications on paper-based charts to electronically documenting them in a standardized format. The informants expressed their frustration and difficulties with the current medication reconciliation process, ordering and administering medicines, and inaccuracies in the medication list. This frustration was particularly evident during patient admission and discharge, as there were numerous medications to reconcile. However, while some informants acknowledged the challenges of entering and managing medication reconciliation in the new EHR system compared to the previous system, they also emphasized the potential for mitigating patient risk. Moreover, some informants expressed contentment with the shift from paper charts to electronic charts for medication lists. Conversely, certain individuals contended that this process was demanding and could potentially increase patient risk:

I find the medication lists to be a bit tangled and unclear, especially when you have to discharge the patient and know what they are actually on, I always feel like there is something that gets overlooked, some medications that are missing or others that have been included but shouldn’t have been, so, the medication lists are challenging.

Informant 1

Furthermore, it was observed that, in some cases, physicians who encountered difficulties with medication ordering or the medication reconciliation process sought assistance from colleagues who were more proficient in using the system.

### Structured and Standardized Clinical Documentation: SNOMED CT and the Use of Templates

#### SNOMED CT: More Nuanced or More Complex Terminology System?

The transition from *ICD-10* to SNOMED CT for documenting patient health conditions was perceived by some informants as challenging and by others as a more nuanced diagnostic system. Challenges usually arose due to difficulties associated with finding the correct diagnosis, as physicians were presented with multiple alternatives when starting to write the name of the diagnosis. In addition, sometimes physicians could not find the diagnosis they needed. When diagnosing with SNOMED CT, physicians could specify the location of a medical condition using the SNOMED CT code:

...in ICD, there is no difference between right and left, or bilaterally, whereas in SNOMED it is easier to...there, the diagnosis code can already indicate differences between right and left, and bilaterally, so it is potentially that I write right and left in the diagnosis code, which I did not do before...

Informant 7

However, in some cases, it was not possible to do this for certain conditions. For instance, during one of the observations, it was noted that when the physician was documenting the diagnosis of deep subfascial lipoma, the physician could not specify the location of the lipoma in SNOMED CT. As a solution, the physician noted the location in the daily notes. The physician pointed out that this information could be essential, especially for surgeons. In addition, the physician mentioned that they could specify the location for a “general” lipoma but not for a subfascial lipoma.

Nonetheless, some informants perceived SNOMED CT as a more nuanced terminology system. Furthermore, SNOMED CT eliminated the need for physicians to commit to memorizing the precise code linked to a specific diagnosis. Instead, they could search for a broader term related to the diagnosis they intended to document. Subsequently, they were presented with multiple alternative options, allowing them to choose the most fitting one.

It is important to note that, in numerous cases, most physicians relied on *ICD-10* codes to identify the corresponding SNOMED CT diagnosis.

#### Use of Templates: Both Limitation and Means of Maintaining Continuity and Quality Assurance

Most informants emphasized the benefits of using templates to document routine procedures, such as the most frequently performed surgeries. They believed that this approach saved time, and, for some informants, it helped them remember all the necessary parameters and documentation requirements:

It goes faster because there is already a lot of text that is already there, and then you just need to fill in the right and left, how long are the screws, what type of plate was used, what kind of things...and the rehabilitation afterward, is it six weeks, or eight weeks...

Informant 7

In addition, it could serve as a checklist for each step involved in a procedure. Many informants had created their own templates, with some developing up to 20 templates for frequently executed operations. However, some of the informants also mentioned that it took time to create these templates properly, so they relied on templates designed by their colleagues. It was also noted that, despite being in a template, these data were not searchable because they were written in free-text format.

Conversely, the informants highlighted that the use of structured and standardized templates for nonstandardized procedures was viewed as both time-consuming and ineffective. It was mentioned that by using a standardized template, physicians might end up documenting unnecessary information, thereby complicating the clinical workflow, particularly during hectic days.

## Discussion

### Principal Findings

This study provides insights into physicians’ documentation practices after they transitioned from an older EHR system to a cross-institutional EHR system, which enables standardized and structured documentation. This transition necessitated physicians to change their documentation habits to some extent, resulting in more clinical data being recorded in a structured and standardized format. However, the degree of acceptance toward new documentation processes is influenced by a range of social and technological factors.

First, according to the study participants, training in system use and ongoing technical support were of utmost importance, particularly after implementing the EHR:

We received a lot of tips and tricks in the beginning, but then it was more about doing, surviving, staying afloat, and stabilizing ourselves a bit...having new training now would have been a much bigger help at this time than it was before...

Informant 1

The importance of training has been well-documented in previous research [64-67]. However, most of the attention and resources allocated for training tend to be centered on the pre–go-live phase and the initial implementation stage of the EHR system, with limited support available for an extended period after the go-live phase and for system updates [65,68,69]. In addition, it has been noted that users may feel overwhelmed during initial training and prioritize familiarizing themselves with the fundamental aspects of the system rather than striving to use it efficiently [68]. This concern was also emphasized by the informants in our study. Moreover, peer-based learning, where physicians are taught by their expert peers in the use of the EHR system, was deemed beneficial. This suggests that acquiring knowledge from fellow experts could prove more advantageous, as these peers possess a deep understanding of the physicians’ workflows and the specific challenges they face when using the system. Peer-based learning has also been observed when physicians use the templates created by their colleagues. Hence, it can be advantageous to create platforms where physicians can share tacit knowledge, enabling them to mutually benefit from their expertise in effectively using the system and implementing best practices for documentation. Providing additional training in documentation practices should be considered, particularly during the transition to the new EHR system, which necessitates changes in physicians’ documentation practices. This training becomes even more significant considering the alarming statistics from a study conducted in Norway. The study, which encompassed 14 hospitals, revealed that in 2003, an incorrect main diagnosis code was assigned in 37.8% of hospital stays, and in 2008, in 36.2% of hospital stays [47]. According to physicians working in these hospitals, the main factors contributing to this issue were challenging code systems (59%) and inadequate training (50%) [70]. The significance of education and ongoing training in good documentation practices as well as its contribution to achieving a learning health system, has been emphasized in numerous research studies [71-75].

Second, in conjunction with the aforementioned first factor, physicians found the transition to the new EHR system to be challenging. The challenge stems from the complex navigation needed to find and execute specific tasks, the overwhelming amount of information in the EHR system, and the inefficient user interface. Furthermore, physicians’ past recording habits in older EHR systems can impact their documentation practices in the new EHR system. As pointed out by Orlikowski [38], when people use technology, “people also draw on their skills, power, knowledge, assumptions, and expectations about the technology and its use, influenced typically by training, communication, and previous experiences” [76]. Given that physicians have established workflows in the previous system, adjusting to new processes in the new EHR system might hinder their adaptability and work efficiency. This finding aligns with previous research conducted by Joukes et al [77], who identified that the preimplementation circumstances played a significant role in shaping the perceived advantages of implementing a new EHR system with structured and standardized clinical data recording. Specifically, the authors emphasized that the perceived impact of the implementation varied depending on whether users were transitioning from older EHR systems or paper-based records. In a paper-based record center, the implementation yielded positive perceived effects on EHR use, data quality, and data reuse. However, in a center that had previously used an EHR system, the perceived effects were predominantly negative or neutral. The authors further highlighted that individuals with previous exposure to EHR systems tended to draw comparisons between the new system and their old one, thereby potentially recognizing that certain tasks were accomplished more efficiently in the former system [77].

While the adoption of the new EHR system has led to an increase in structured and standardized data recording, it is often not a decision made by physicians but rather an imposition by the new system and health organization. In certain cases, the structured and standardized documentation does not align with physicians’ traditional workflow; therefore, it takes more time compared to free-text documentation. Moreover, physicians occasionally experience uncertainty and difficulties regarding the correct way to document structured and standardized data or question their necessity. This may potentially lead to inconsistencies in data recording. Data that could be standardized may instead be documented in a free-text format, and when faced with time constraints, physicians may use work-arounds, such as quickly clicking through to proceed further. This practice might also explain physicians’ distrust toward some of the standardized clinical data available in the EHR system, such as problem lists and medical history. One advantage of the problem list and medical history is that these remain visible to physicians whenever they access patient records, without the need to open journal notes. While this provides better information at the point of care, the problem lists and medical history lose their advantage if physicians fail to document or update it or if they lack trust in the accuracy and reliability of this information. Moreover, the prevalent reliance of physicians on the *ICD-10* codes to identify the corresponding SNOMED CT codes may undermine the advantages of using a more comprehensive terminology system, SNOMED CT. This reliance can lead to loss of clinical detail and mapping inaccuracies. This practice indicates a discrepancy between the physicians’ mental models (understanding and reasoning about systems) and the designer’s conceptual model (how the system is intended to be used) [78,79]. Mental models, derived from a range of factors, including experiences, knowledge, and perception, have been widely applied in the field of human-computer interaction research [79,80]. Gaining an understanding of physicians’ mental models can be important for effectively optimizing the system and tailoring training in documentation practices in the new EHR system. The greater the disparity between the designer’s conceptual model and the user’s mental model, the more challenging it becomes for individuals to effectively use the system [78,79]. Furthermore, this emphasizes the need to distinguish between technologies as artifacts and technologies in practice (how technology is actually used rather than solely focusing on the technology itself), as it is only through effective use that technology can impact productivity [38]. Consequently, when enhancing technology, it becomes vital to consider sociotechnical factors, which, in turn, necessitates the technology to possess sufficient flexibility in order to be customized according to specific local needs and evolving requirements [33,37]. Moreover, Rosenbloom et al [22] have underscored the significance of offering flexibility in the documentation processes, as they are highly valued and essential for health care professionals. By using free-text documentation, health care professionals can document intricate and nuanced details about a patient, which can also prove to be more valuable for subsequent health care professionals taking over the patient’s care [22]. Some of our study participants have also emphasized that reading high-quality free-text journal notes can provide them with more crucial information compared to solely reviewing standardized or structured data:

The only way I manage to get an overview of the medical history of a patient is basically if someone has written something reasonable in a journal note.

Informant 4

Having the right balance between standardization and flexibility in documentation processes is crucial, particularly in situations where unforeseen clinical findings or unexpected circumstances arise [22].

According to the study participants, most physicians have a positive attitude toward using templates for documenting routine procedures:

It goes faster [using templates] because there is already a lot of text that is already there, and then you just need to fill in the right and left, how long are the screws, what type of plate was used, what kind of things...and the rehabilitation afterward, is it six weeks, or eight weeks...

Informant 7

While certain physicians have created their own templates for frequently conducted surgeries, others depend on templates designed by their peers. However, the information contained within these templates is recorded in a free-text format. As a result, these data are not conducive to analysis using statistical or machine learning techniques. Moreover, when physicians develop their own templates for commonly performed surgeries, it can result in inconsistencies in documentation and potential omissions of critical data that need to be documented. On the other hand, this behavior might be elucidated through the analysis of physician-patient dialogues in Norwegian general practice by Nessa [81,82]. His research emphasized that these conversations were a fundamental aspect of a physician’s work, encompassing rich narratives and certain elements such as context and emotional tone [81,82]. Such complexities may be challenging to capture within a standardized template and encoded data. Thus, adhering strictly to encoded data and standardized templates implied by the organization may impose a semiotic burden on physicians, requiring them to exert considerable mental effort to fit complex narratives into standardized templates. The attitudes of health care professionals toward structured and standardized documentation may also depend on their perception of the benefits it provides [21,83]. Our study participants also highlighted this aspect (eg, saving time in postoperative documentation), suggesting that physicians may view the implementation of standardized or semistandardized templates for commonly performed procedures, developed in collaboration with physicians and following established guidelines, as a positive step. Moreover, this approach could potentially minimize variations in clinical documentation. This approach might also enable more data to be consistently recorded in a standardized format and enhance guideline compliance. Several studies have shown that the use of templates improves the accuracy of documentation [84-86]. For instance, Thomson et al [85] demonstrated the advantages of using a standardized operative note template adhering to established guidelines. This implementation led to enhanced documentation quality and a decrease in the missing data.

### Limitations and Future Research

One limitation of our study is that it involved a limited number of physicians, and data were collected from only 1 hospital. Therefore, the findings of this study may only reflect physicians’ perspectives working within this specific context. Nonetheless, we have made an effort to gather information on documentation practices that can be applied more broadly. Specifically, our focus was on understanding documentation practices when physicians transition from an older EHR system to a new EHR system that facilitates structured and standardized documentation. In addition, we aimed to identify the factors influencing physicians’ choice of documentation methods, such as free text and standardized documentation, regardless of the EHR system they use. Thus, our findings may have relevance beyond the specific organization and the EHR system used in this study, providing insights that can be applied to a wider context.

Moreover, although it is evident that the implementation of the new EHR system has increased the amount of clinical data recorded in a standardized format, our study did not objectively evaluate either the quality of these data (eg, accuracy and completeness) or their effectiveness for secondary uses. Consequently, future research should aim to objectively assess the quality of the data. Furthermore, additional investigations could delve into the perceived data quality from the perspectives of individuals using the data from the new EHR system for secondary purposes, such as quality assurance. This would help determine whether the transition to a more standardized EHR system has positively impacted the use of these data for secondary purposes.

### Conclusions

The findings of this study identified that physicians’ acceptance of new documentation processes is influenced by a range of social and technological factors. These factors include physicians’ past experiences with documentation, perceived benefits, familiarity with the EHR system, and time constraints, all of which can impact their choice of documentation methods. Furthermore, it is important to consider the user-friendliness of the EHR system and how well the documentation processes align with physicians’ workflows. To facilitate the transition to a new EHR system, health care organizations should provide tailored training programs on documentation practices. This is particularly crucial after the implementation of the new system. In addition, it would be advantageous to establish platforms that allow physicians to exchange tacit knowledge and expertise on effectively using the system. Moreover, physicians highly value flexibility in clinical documentation. Therefore, health care organizations should consider implementing standardized documentation processes that also align with physicians’ preferences and allow them some flexibility in the documentation process. For instance, introducing common standardized or semistandardized templates for routine procedures, developed in collaboration with physicians, could potentially minimize disparities in clinical documentation, ensure the consistent recording of data, and improve adherence to guidelines.

## Appendix

Multimedia Appendix 1 Semistructured interview guide.

Multimedia Appendix 2 SRQR (Standards for Reporting Qualitative Research) checklist.

## Abbreviations

EHR: electronic health record
ICD-10: International Classification of Diseases, 10th Revision
SNOMED CT: Systematized Nomenclature of Medicine–Clinical Terms
SRQR: Standards for Reporting Qualitative Research

## Data Availability

The datasets generated or analyzed during this study are not publicly available to protect participant privacy but are available from the corresponding author on reasonable request.
