# Supplementary material for: Exploring Physicians’ Dual Perspectives on the Transition From Free Text to Structured and Standardized Documentation Practices: Interview and Participant Observational Study
Source: JMIR Form Res. 2025 Mar 21;9:e63902. doi: 10.2196/63902 (PMC11971576; doi:10.2196/63902)
Supplement: Multimedia Appendix 1 [file formative_v9i1e63902_app1.docx]

**Semi-structured interview guide**

**Personal data**

1. How old are you?
2. When did you finish your medical education?
3. Specialization (if any)?
4. Can you describe your current position/role at the hospital?
5. Do you usually work in multiple healthcare facilities? For example, inpatient ward, emergency room, etc.
6. Previous experience with EHR (which types of EHR systems have you used before)?

**Training in/experience with the new EHR system**

1. How long have you been using the new EHR?
2. How often do you use the new EHR in your work?
3. Can you tell me a bit about the training you have received in the use of the new EHR?
4. How do you evaluate the training in the use of the EHR, particularly concerning clinical documentation?
5. Are there aspects of the training that you believe could have been improved or done differently?

**General questions about medical doctors' experience with EHR documentation**

1. What kind of information do you document in the EHR?
2. Your EHR system has recently been changed. How does this impact your daily documentation process?

                       a. What has become easier than before?

b. What has become more difficult?

1. When do you prefer to document patient data/clinical work (prior to, during, or after the patient's visit)?
2. Can you think of any modifications to the EHR that would simplify the documentation process?
3. What is your opinion on the purpose of documentation?

**Documentation method (free text/****standardized documentation)**

1. When documenting patient information, which format do you prefer (free text, standardized/structured format (SNOMED CT/ templates))?

a. Why do you prefer this method of documentation?

b. What are the benefits and challenges of your preferred documentation method?

1. Can you describe the types of information you typically document using free text versus standardized documentation?
2. Which factors impact your choice of documentation format?
3. What do you think about using SNOMED CT to document patient diagnoses and procedures?
4. Has SNOMED CT influenced your documentation practice? If so, how?

**Templates**

1. Do you have experience using templates?
   - - *Do you use templates for post-operative description, for the procedures you perform, etc.?*
2. What advantages or disadvantages do you see in using templates for clinical documentation?
3. Do you usually create or use your own templates for documenting clinical data? If yes, what is the reason for that?

Is there anything important about clinical documentation that we haven't discussed?

Thank you for your participation!
