# Supplementary material for: Exploring Physicians’ Dual Perspectives on the Transition From Free Text to Structured and Standardized Documentation Practices: Interview and Participant Observational Study
Source: JMIR Form Res. 2025 Mar 21;9:e63902. doi: 10.2196/63902 (PMC11971576; doi:10.2196/63902)
Supplement: Multimedia Appendix 2 [file formative_v9i1e63902_app2.docx]

| No | Topic | Item |
| --- | --- | --- |
| Title and anbstract | | |
| S1 | Title | Page 1 |
| S2 | Abstract | Page 2 |
| Introduction | | |
| S3 | Problem formulation | Page 3-4 and Page 6 |
| S4 | Purpose or research question | Page 4 |
| Methods | | |
| S5 | Qualitative approach and research paradigm | Page 7 |
| S6 | Research characteristics and reflexivity | Page 10 |
| S7 | Context | Page 6-7 |
| S8 | Sampling strategy | Page 7 |
| S9 | Ethical issue pertaining to human subjects | Page 10-11 |
| S10 | Data collection methods | Page 8-9 |
| S11 | Data collection instruments and technologies | Page 8-9 |
| S12 | Units of study | Page 7-8 |
| S13 | Data processing | Page 8-10 |
| S14 | Data analysis | Page 9-10 |
| S15 | Techniques to enhance trustworthiness | Page 9-10 |
| Results and findings | | |
| S16 | Synthesis and interpretation | Page 11-17 |
| S17 | Links to empirical data | Page 12-17 |
| Discussion | | |
| S18 | Integration with prior work, implications, transferability, and contribution (s) to the field | Page 17-21 |
| S19 | Limitations | Page 21-22 |
| Other | | |
| S20 | Conflict of interest | Page 23 |
| S21 | Funding | Page 23 |

Table1. SRQR -standards for reporting qualitative research
